# The effects of reimbursement reform of antidiabetic medicines from the patients’ perspective – a survey among patients with type 2 diabetes in Finland

**DOI:** 10.1186/s12913-019-4633-9

**Published:** 2019-10-29

**Authors:** Taika Suviranta, Johanna Timonen, Janne Martikainen, Emma Aarnio

**Affiliations:** School of Pharmacy, University of Eastern Finland, P.O. Box 1627, 70211 Kuopio, Finland

**Keywords:** Reimbursement reform, Antidiabetic medicines, Type 2 diabetes, Survey

## Abstract

**Background:**

In Finland, the reimbursement rate for antidiabetic medicines other than insulins was lowered from 100 to 65% at the beginning of 2017. The objective of this study was to examine the effects of this reform experienced by patients with type 2 diabetes. The objective was also to explore if socio-economic status affects this experience.

**Methods:**

The data were collected by conducting a survey among Finnish adults with type 2 diabetes (*n* = 603). The baseline survey was conducted in November–December 2016. A second follow-up survey was conducted at the end of 2017 where the participants’ experience of the reimbursement reform was surveyed with an open-ended question. Free-form inductive content analysis was used to categorize the answers. The association between the participants’ characteristics and reporting an effect caused by the reimbursement reform was studied with binomial logistic regression.

**Results:**

285 (47.3%) participants reported an effect of some kind caused by the reimbursement reform. The most common reported effects were economic effects (32.7%) and annoyance (12.4%). Having financial difficulties in purchasing antidiabetic medicines (odds ratio (OR) 5.20, 95% confidence interval (Cl) 2.99–9.06) or not having annual deductible exceeded (OR 2.17, 95% CI 1.19–3.95), and use of certain antidiabetic medication groups at baseline were associated with reporting an effect. Socio-economic status was not associated with the likelihood of reporting an effect.

**Conclusions:**

Almost half of the participants with type 2 diabetes reported an effect, most commonly economic effects, such as increased expenditure or difficulty in purchasing medicines, after the reimbursement reform. It is important to study the effects of reimbursement reforms also from the patients’ perspective.

## Background

Diabetes is a progressive disease defined by chronically elevated blood glucose levels [1]. There has been continuing growth in rates of diabetes incidence and prevalence worldwide [2]. Diabetes imposes human, social and economic burden. For example, over 500,000 Finns (total population 5.5 million) have diabetes and its treatment costs cover about 15% of the total expenditure of Finnish health care which was EUR 20.6 billion in 2017 [3, 4].

**Fig. 1:**
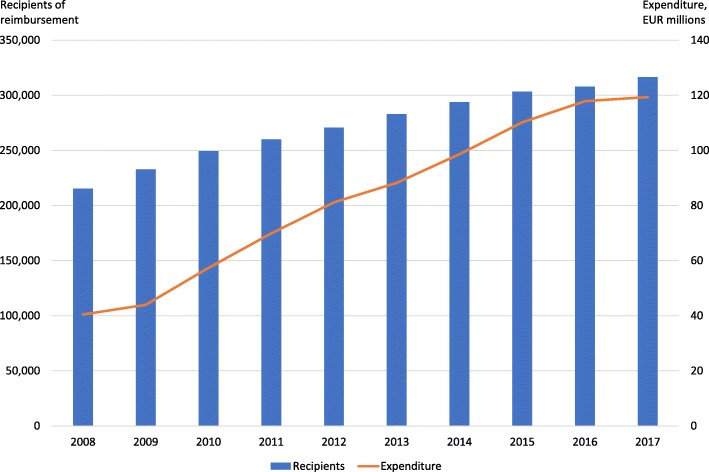
Recipients of reimbursement and expenditure on blood glucose lowering medicines, excluding insulins (ATC-code A10B) in Finland [6]

Antidiabetic medicines are a significant part of the management of diabetes as they can improve health outcomes and quality of life [2]. Metformin is recommended as the first line medication for type 2 diabetes [3, 5]. Other older oral medicines for type 2 diabetes include sulfonylureas, glitazones, and glinides which all can cause weight gain. Sulfonylureas and glinides can also cause hypoglycemia. In the 2000’s, glucagon-like peptide-1 (GLP-1) -analogues, dipeptidyl peptidase-4 (DPP-4) -inhibitors, and sodium-glucose co-transporter 2 (SGLT2) -inhibitors have entered the market. In addition to their glucose-level lowering effects, use of GLP-1-analogues, which are injectable, and SGLT2-inhibitors is associated with weight loss. Expenditure of antidiabetic medicines, especially other than insulins, has increased quickly during the last years in Finland (Fig. 1) [6, 7]. Growth in expenditure resulted from an increase in the number of patients and a change towards newer and more expensive antidiabetic medicines [7].

In 2015, the Finnish government set an aim of EUR 150 million savings in medicine reimbursement expenditure [8]. According to the suggestions in a report by an appointed examiner [9], the reimbursement rate for antidiabetic medicines other than insulins was lowered from 100 to 65% in Finland at the beginning of 2017 (the Finnish reimbursement system and the reimbursement reform are described in the Settings). The reimbursement reform saved about EUR 26 million in reimbursement expenditure and increased copayments paid by patients considerably in 2017 [10, 11]. During the preparation of the reimbursement reform there was discussion about increased copayment leading to impaired therapeutic control or difficulties in purchasing medicines [7]. According to the statistics on medicines, the reimbursement reform has not affected the consumption of antidiabetic medicines significantly [10, 11]. In 2017, the consumption of antidiabetic medicines other than insulins decreased by 1% from the preceding year while the number of patients receiving reimbursement for those medicines increased by 3%. However, previous studies have proved that higher copayment can be associated with poorer adherence to antidiabetic medicines [12–17]. Moreover, before the implementation of the reimbursement reform, it was estimated that copayments would increase more often among low-income patients [7]. This was due to the fact that the majority of Finnish patients with type 2 diabetes are pensioners and pensioners are more often in the lower income categories than those of working age.

Previous studies have shown that reimbursement reforms generally have effects on the utilization, adherence, expenditures or prescription patterns of antidiabetic medicines [18–22]. To our best knowledge, the effects have not been studied from the patients’ perspective before. Previous studies have been based on register data, and consequently they do not provide information from the patients’ point of view [18–22]. The aim of this study was to investigate the effects experienced by patients with type 2 diabetes regarding the reimbursement reform of antidiabetic medicines in Finland. The aim was also to explore if socio-economic status affects this experience.

## Methods

### Setting

#### Medicine reimbursement system in Finland

According to the Health Insurance Act, the medicine reimbursement system is administered by the Social Insurance Institution of Finland (Kela) [23]. All Finnish residents are eligible for reimbursement for medicines which are used for the treatment of an illness. Reimbursements are available after the Pharmaceuticals Pricing Board (Hila) has approved the reimbursement status of the medicine, basic topical ointment, or clinical nutritional preparation and confirmed its reasonable wholesale price. The prices of medicines are the same in every pharmacy and customers usually receive reimbursement directly at the pharmacy. Medicines can be reimbursed up to a three months’ supply at one transaction. There is also a reference price system in use in Finland, which is based on generic substitution.

The medicine reimbursement system consists of three categories: the basic rate (40%), the lower special rate (65%) and the higher special rate of reimbursement (100%) [23]. The rate of reimbursement depends on disease severity and the necessity of the medicine treatment. Primarily, the basic rate of reimbursement is paid to all individuals covered by the Finnish Health Insurance Scheme, if a reimbursement status is approved for the medicine. The government decrees the diseases that entitle medicinal products to be reimbursed at the special rate. The lower special rate of reimbursement covers serious and chronic diseases, for example arterial hypertension and bronchial asthma. The category of the higher special rate of reimbursement consists of serious and chronic diseases where the medicine restores or replaces normal bodily functions and where the medicine treatment is necessary for the patient, for example epilepsy, glaucoma and breast cancer. The purchase price or reference price of a medicinal product belonging to the higher special rate of reimbursement is reimbursed to the customer in full. However, the customer pays a copayment of EUR 4.50 per each medicinal product.

Reimbursement is paid after the annual sum of reimbursable medicinal products paid by the customer exceeds the initial deductible, EUR 50 [23]. The initial deductible is applicable from the beginning of the year in which a customer reaches the age of 19 years. There is also a limit for the annual maximum on out-of-pocket costs (i.e., annual deductible). After the annual deductible is exceeded, the customer is entitled to an additional reimbursement, which means that the customer pays EUR 2.50 copayment per transaction of each reimbursable medicine. In 2017, the limit of annual deductible was EUR 605.13.

#### Reimbursement reform of antidiabetic medicines

Several changes were conducted to the medicine reimbursement system in 2017 to achieve the total savings of EUR 150 million in reimbursement expenditure as required by the Government Programme [8]. As suggested in the report by the examiner appointed by the Ministry of Social Affairs and Health [9], one of the changes was the lowering of the reimbursement rate of antidiabetic medicines. The reimbursement rate for antidiabetic medicines other than insulin products was lowered from higher special rate (100%) to lower special rate (65%) of reimbursement. The reimbursement rate of insulin treatment remained at the higher special rate for all diabetic patients because it is a replacement therapy. The reimbursement rate for type 2 diabetes medicines was transferred into the same reimbursement category as medicines for cardiovascular diseases. According to the government proposal, this was reasonable, because lifestyle changes are essential in treatment and prevention of type 2 diabetes as in treatments of coronary artery disease and arterial hypertension [7]. Moreover, the reimbursement reform was aimed at antidiabetic medicines because their expenditure had increased constantly (Fig. 1).

The effect of the reimbursement reform on copayments paid by patients was estimated beforehand based on previous purchases of antidiabetic medicines [24]. For patients using DPP-4-inhibitors or GLP-1-analogues, the annual copayment increase was estimated to be EUR 157 on average. The corresponding figure for patients using older antidiabetic medicines (e.g., metformin and sulfonylureas) was EUR 12. For some patients, the increase in annual copayments was estimated to be over EUR 300.

### Data collection

The study data were collected by conducting a survey among Finnish adults with type 2 diabetes. The aim of the survey was to find out how the reimbursement reform has affected the use of medicines, treatment outcomes, and satisfaction with care among patients with type 2 diabetes. The participants were recruited at pharmacies across Finland using convenience sampling among eligible customers. Pharmacists were instructed to recruit adult patients with type 2 diabetes who used medication to lower blood glucose. Patients were not required to be buying medicines when recruited. Patients with type 1 diabetes or gestational diabetes were excluded as well as patients with double diabetes using only insulin. Pharmacists received training for the recruitment. The pharmacies involved in this study were part of a research pharmacies’ network. The participants received an announcement of the study before they agreed to participate.

The data collection included three stages: baseline survey, 6-month follow-up survey and 12-month follow-up survey. In this article, material from the baseline and 12-month follow-up survey is used. The baseline survey was conducted before the implementation of the reimbursement reform. Nine hundred fifty-five participants from 114 pharmacies replied to the baseline survey in November–December 2016 with mobile tablet devices in pharmacies. These 114 pharmacies were of different size and located all around Finland. Follow-up surveys were replied by phone interviews or electronic survey depending on the participant’s preference. A link via text message or e-mail was sent to those choosing to answer the electronic survey. Phone interviews were performed by several interviewers who filled the survey electronically on behalf of the respondent (i.e., interviews were not recorded). The data collected by phone interviews and electronic survey were combined.

In this article, the answers for one question which was added to the 12-month follow-up survey in November–December 2017 are being reported. The participants’ experience of the reimbursement reform was surveyed with an open-ended question: “*How has the reimbursement reform implemented in 2017 concerning antidiabetic medicines affected your life?*”. In the electronic survey, participants were instructed to skip the question if the reform had not affected their life. In the surveys, the participants’ sociodemographic and socio-economic characteristics (gender, age, household’s monthly income, education, working situation), use of antidiabetic medicines (medicines used, daily dosages, length of use), diabetic complications, contacts with health care, satisfaction with their diabetes care, details about their disease, and medication copayments were surveyed with structured questions.

### Data analysis

The data analysis was two phased and included qualitative and quantitative analysis. In the qualitative analysis, the data were first analyzed using free-form inductive content analysis which aim is to gain condensed description of the data systematically and objectively [25]. The analysis started by reading through the answers to become familiar with the data as a whole. The analysis unit could be a single word, a sentence or a group of sentences describing an idea relating how the reimbursement reform has affected the participant’s life. Then simplifications were formed from answers, one answer including possibly multiple simplifications (Table 1). The simplifications were compared and sorted into emerging subcategories. The subcategories were named so that the name described all the simplifications in the subcategory. Similar subcategories were unified to main categories, named similarly as mentioned above. Free-form inductive content analysis was conducted by one researcher (TS) using Word 2016 (Microsoft Corporation, Redmond, WA). The analysis was discussed with other research group members. All the presented quotes are from the electronic survey.

**Table 1:** Examples of categorization of the answers

Original phrase	Simplification	Subcategory	Main category
*“Finances are tighter than before.”*	Economic situation has worsened	Increased expenditure	Economic effects
*“I use less money on other expenses.”*	Had to save on other costs	Purchasing medicines has required saving or borrowing money	Economic effects
“*The medicine that has become more expensive has to be bought for a month at a time”*	Cannot buy 3 months’ supply of medicines at a time	Difficulty of purchasing medicines	Economic effects
*“I had to, against doctor’s orders, quit the medications. Both [GLP-1-analogue] and [SGLT2-inhibitor] because of the financial situation.”*	Use of medicines has discontinued	Effects on use of medicines	Effects on use of medicines
*“I’m careful and might sometimes skip taking a pill if the blood sugar level has been good.”*	Has not taken the medicine if blood sugar level has been good	Effects on use of medicines	Effects on use of medicines
*“My long-term blood sugar has worsened significantly.”*	Therapeutic control has worsened	Impaired therapeutic control	Effects on health

After the categorization of the answers, the association between the participant characteristics at baseline (survey 2016) and reporting an effect caused by the reimbursement reform (12-month follow-up survey 2017) was studied with binomial logistic regression with reporting any effect as the dependent variable (vs. not reporting any effect). The following sociodemographic and socio-economic characteristics were included in the analysis: age, gender, household’s monthly income (less than EUR 1000, EUR 1000–1999, EUR 2000–2999, EUR 3000–3999, EUR 4000 or more), education level (basic education or some other/vocational upper secondary education and training/post-secondary non-higher vocational education/matriculation examination/university or polytechnic degree), working situation (working vs. not), financial difficulties in purchasing antidiabetic medicines, and whether the annual maximum limit on out-of-pocket costs was exceeded. Diabetes-related characteristics included the participants’ disease history, number of diabetic complications, use of different antidiabetic medication groups, and use of hypertension and cholesterol medication. One researcher (EA) performed statistical analyses using SAS version 9.4 (SAS Institute, Inc., Cary, NC). The comparability of the survey participants with Finnish patients with type 2 diabetes was examined using publicly available data on entitlements to reimbursement and recipients of reimbursement [6].

### Ethical statement

The study complied with the national ethical principles of research [26]. According to instructions, this study did not require ethical approval. Participation in the study was voluntary and answering was regarded as an informed consent to participate in the survey. Researchers had access only to de-identified data.

## Results

**Fig. 2:**
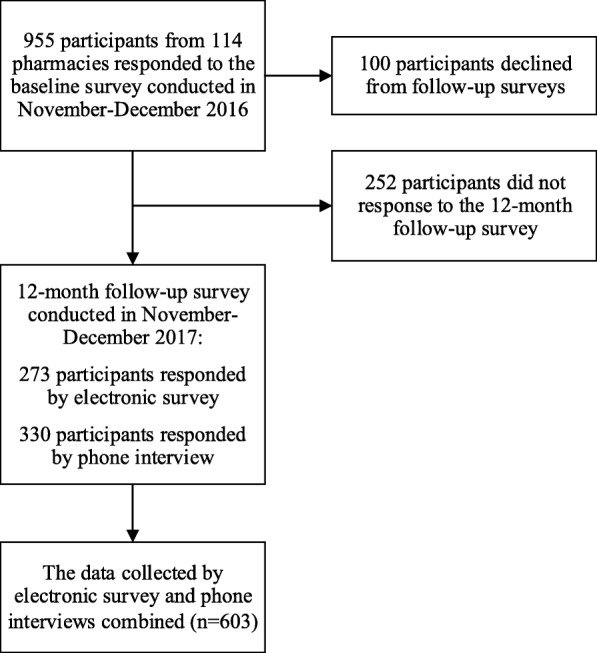
Study flow

Six hundred three participants responded to the 12-month follow-up survey conducted in November and December 2017 (Fig. 2). The patients dropping out of the 12-month follow-up survey were more often working than the participants (Additional file 1). 285 (47.3%) participants reported some kind of effect caused by the reimbursement reform. In total, 318 (52.7%) participants did not report any effect on life including 75 (12.4%) participants responding that the reimbursement reform had not affected their life. The remaining 243 participants did not respond to the question. Because in the electronic survey participants were instructed to skip the question in case the reform had had no effect, they were categorized as not having reported any effect.

The characteristics of the participants are shown in Table 2. Almost all participants used other antidiabetic medicines than insulin at the time of the baseline survey (Table 2). From other antidiabetic medicines than insulin, 210 participants (34.8%) used only metformin at baseline. The majority of participants were not working or were outside working life. Only 17% of the participants had completed the matriculation examination or a university or polytechnic degree. Compared to patients with type 2 diabetes in Finland, 60–69-year-olds seemed to be overrepresented and over 80-year-olds underrepresented (Additional file 2). When comparing the use of antidiabetic medicines other than insulin, survey participants corresponded well, on average, to Finnish patients with type 2 diabetes (Additional file 3). However, the use of GLP-1-analogues and SGLT2-inhibitors seemed to be slightly more common among survey participants.

**Table 2:** Baseline characteristics of the survey participants

	Effect reported (*n* = 285) % (n)	No effect reported (*n* = 318) % (n)	All (*n* = 603) % (n)
Sociodemographic and -economic variables			
Mean age*, years (SD)	64.4 (9.4)	66.5 (10.4)	65.5 (10.0)
Female gender	48.1 (137)	49.1 (156)	48.6 (293)
Household’s monthly income			
Less than EUR 1000	11.6 (33)	11.0 (35)	11.3 (68)
EUR 1000–1999	40.0 (114)	34.6 (110)	37.1 (224)
EUR 2000–2999	27.7 (79)	29.6 (94)	28.7 (173)
EUR 3000–3999	10.9 (31)	11.6 (37)	11.3 (68)
EUR 4000 or more	9.8 (28)	13.2 (42)	11.6 (70)
Education			
Basic education or some other	40.7 (116)	38.7 (123)	39.6 (239)
Vocational upper secondary education and training	21.4 (61)	19.8 (63)	20.6 (124)
Post-secondary non-higher vocational education	19.6 (56)	25.5 (81)	22.7 (137)
Matriculation examination	5.6 (16)	5.7 (18)	5.6 (34)
University or polytechnic degree	12.6 (36)	10.4 (33)	11.4 (69)
Work/life situation			
Working	14.0 (40)	18.6 (59)	16.4 (99)
Not working or outside working life	86.0 (245)	81.4 (259)	83.6 (504)
Financial difficulties in purchasing antidiabetic medicines*	28.8 (82)	7.9 (25)	17.7 (107)
Annual maximum limit on out-of-pocket costs exceeded			
Yes	10.5 (30)	13.8 (44)	12.3 (74)
Will be exceeded	6.0 (17)	6.3 (20)	6.1 (37)
Will not likely be exceeded or does not know	83.5 (238)	79.9 (254)	81.6 (492)
Diabetes-related variables			
How long has had diabetes*			
More than 20 years	11.6 (33)	8.5 (27)	10.0 (60)
11–20 years	27.4 (78)	26.4 (84)	26.9 (162)
6–10 years	37.5 (107)	28.3 (90)	32.7 (197)
2–5 years	21.4 (61)	26.4 (84)	24.0 (145)
1 year or less	2.1 (6)	10.4 (33)	6.5 (39)
Mean number of diabetes complications (SD)	0.8 (1.1)	0.8 (1.1)	0.8 (1.1)
Use of insulin	33.3 (95)	28.6 (91)	30.8 (186)
Use of other antidiabetic medicines than insulin	98.6 (281)	96.5 (307)	97.5 (588)
Metformin	74.7 (213)	76.7 (244)	75.8 (457)
Sulfonylureas	4.6 (13)	2.8 (9)	3.6 (22)
Combination of oral blood glucose lowering medicines*	16.1 (46)	9.1 (29)	12.4 (75)
Glitazones*	4.2 (12)	1.3 (4)	2.7 (16)
DPP-4-inhibitors*	44.9 (128)	29.6 (94)	36.8 (222)
Glinides	1.1 (3)	0.9 (3)	1.0 (6)
GLP-1-analogues*	13.0 (37)	6.3 (20)	9.5 (57)
SGLT2-inhibitors*	21.4 (61)	7.5 (24)	14.1 (85)
Use of hypertension medication	81.4 (232)	78.0 (248)	79.6 (480)
Use of cholesterol medication	70.2 (200)	65.4 (208)	67.7 (408)

*statistically significant difference (*p* < 0.05) between participants who reported an effect and who did not

*DPP-4* Dipeptidyl peptidase-4, *GLP-1* Glucagon-like peptide-1, *SD* Standard deviation, *SGLT2* Sodium-glucose co-transporter 2

Five main categories and eight subcategories were identified from the participants’ answers (Table 3). Economic effects was the most common main category.

**Table 3:** The main categories and subcategories found in the study (N = 603)

Category	n^a^	%
Economic effects	197	32.7
Increased expenditure	105	17.4
Difficulty in purchasing medicines	57	9.5
Purchasing medicines has required saving or borrowing money	52	8.6
Annoyance^b^	75	12.4
Effects on use of medicines^b^	46	7.6
More accurately undefined effect^b^	13	2.2
Effects on health	8	1.3
Impaired therapeutic control	6	1.0
Impaired quality of life	2	0.3

^a^One answer could include multiple main categories and/or subcategories

^b^Includes only one subcategory

### Economic effects

197 (32.7%) participants reported economic effects, which included three subcategories (Table 3). One hundred five participants reported that their expenditure had increased. This involved in most cases increased medicine expenditure (*n* = 43) or worsened economic situation (*n* = 27). Some reported that the reimbursement reform had decreased the amount of money they have available (*n* = 12) and some replied that their spending had become more careful (*n* = 10).

*“have to carefully think about the money the month you have to buy the medicine”* [63-year-old female]

Fifty-seven participants’ responses indicated difficulty in purchasing medicines. Difficulties were related to, for example, not being able to buy three months’ supply of medicines at a time (*n* = 14) and some had to consider when they can purchase medicines (*n* = 14). Ten participants feared that purchasing medicines might become more difficult in the future

*“As someone with a small income it’s a constant problem to get enough money to buy medicines.”* [47-year-old male]

Purchasing medicines had required saving or borrowing money for 52 participants. In most cases, participants had to save on food (*n* = 14) or other costs (*n* = 28). Seven participants had had to borrow money to purchase medicines.

*“it tightens other buying power, we buy cheaper food and maybe not as healthy.”* [57-year-old female]

### Annoyance

75 (12.4%) participants reported that the reimbursement reform annoyed them (e.g., resentment, anger or criticism towards the reform). Of them, 15 reported that the reform had not had an effect on their personal life, but still felt annoyed by it.

*“Still have been able to buy [medicines]. The most stupid decisions that have been made!”* [51-year-old male]

### Effects on use of medicines

46 (7.6%) participants described effects on use of medicines. 14 participants reported that they had discontinued using a medicine. Four participants had considered to discontinue their medicine use. Some had had breaks from using a medicine (*n* = 7) and some had decreased their medicine use (*n* = 4). Two participants reported that they had switched to insulin, which remained at the highest rate of reimbursement. Four participants had considered switching to insulin and two participants told that their level of insulin had increased.

*“Medication had to be changed completely because medical expenses increased heavily.”* [61-year-old female]

### More accurately undefined effect

13 (2.2%) participants reported that the lowering of the reimbursement rate had affected their lives, but they had not described the effect more accurately.

*“it did affect indeed …*” [64-year-old female]

### Effects on health

Eight (1.3%) participants reported effects on health. Six participants reported that their therapeutic control had impaired due to the reimbursement reform. Two participants brought forward impaired quality of life.

*“It has affected a great deal, I can’t buy [GLP-1-analogue] that would allow me to improve my blood sugar values.”* [63-year-old female]

### Associations between participants’ baseline characteristics and reporting an effect

Based on the performed logistic regression, gender, income, work status, or education level at baseline were not associated with the likelihood of reporting an effect (Table 4). Older people were less likely to report an effect (odds ratio (OR) 0.97 per year, 95% confidence interval (CI) 0.94–0.99). Also, participants who had had diabetes for only a year or less were less likely to report an effect (OR 0.17, 95% CI 0.05–0.55) when compared to participants who had had their diagnosis more than 20 years ago. Having financial difficulties in purchasing antidiabetic medicines (OR 5.20, 95% CI 2.99–9.06) or not having the annual deductible exceeded (OR 2.17, 95% CI 1.19–3.95) and the use of certain antidiabetic medication groups (combinations of oral blood glucose lowering medicines, glitazones, DPP-4-inhibitors, GLP-1-analogues, SGLT2-inhibitors) at baseline were associated with reporting an effect.

**Table 4:** Adjusted odds ratios of reporting an effect caused by the reimbursement reform

	Any effect
	OR (95% CI)
Sociodemographic and -economic variables	
Age	0.97 (0.94–0.99)
Female gender	1.13 (0.77–1.66)
Household’s monthly income	
Less than EUR 1000	1.00
EUR 1000–1999	1.15 (0.59–2.23)
EUR 2000–2999	0.95 (0.48–1.88)
EUR 3000–3999	1.05 (0.45–2.46)
EUR 4000 or more	0.86 (0.35–2.08)
Education	
Basic education or some other	1.00
Vocational upper secondary education and training	1.14 (0.68–1.92)
Post-secondary non-higher vocational education	0.70 (0.41–1.18)
Matriculation examination	0.99 (0.41–2.43)
University or polytechnic degree	1.28 (0.65–2.52)
Working	0.62 (0.33–1.14)
Financial difficulties in purchasing antidiabetic medicines	5.20 (2.99–9.06)
Annual maximum limit on out-of-pocket costs exceeded	
Yes	1.00
Will be exceeded	1.33 (0.52–3.41)
Will not likely be exceeded or does not know	2.17 (1.19–3.95)
Diabetes-related variables	
How long has had diabetes	
More than 20 years	1.00
11–20 years	0.60 (0.30–1.20)
6–10 years	0.82 (0.41–1.66)
2–5 years	0.63 (0.29–1.35)
1 year or less	0.17 (0.05–0.55)
Number of diabetes complications	0.91 (0.76–1.10)
Use of antidiabetic medicines	
Insulin	0.97 (0.62–1.52)
Metformin	1.41 (0.84–2.35)
Sulfonylureas	1.37 (0.50–3.76)
Combinations of oral blood glucose lowering medicines	4.26 (2.13–8.53)
Glitazones	3.87 (1.15–13.01)
DPP-4-inhibitors	2.93 (1.89–4.53)
Glinides	1.12 (0.21–5.95)
GLP-1-analogues	3.47 (1.76–6.83)
SGLT2-inhibitors	2.44 (1.39–4.26)
Use of hypertension medication	1.30 (0.80–2.13)
Use of cholesterol medication	1.18 (0.77–1.81)

*CI* Confidence interval, *DPP-4* Dipeptidyl peptidase-4, *GLP-1* Glucagon-like peptide-1, *OR* Odds ratio, *SGLT2* Sodium-glucose co-transporter 2

## Discussion

In our study, around 47% of the participants reported an effect of some kind caused by the reimbursement reform of antidiabetic medicines. Participants reported most commonly economic effects, including increased medicine expenditure, worsened economic situation, difficulties in purchasing medicines or need to save on other costs. This type of study provides valuable information that cannot be detected with register data. For instance, according to statistics on medicines, the reimbursement reform has not significantly reduced the consumption of antidiabetic medicines even though patients’ copayments increased [10, 11]. However, some participants reported operational changes in their lives which does not appear in registers, such as the need to save or borrow money to purchase medicines.

Most of the participants not reporting any effect did not respond to the question. The analyzed open-ended question was the last question of a relatively long questionnaire, so it is possible that some of the participants passed the question because of a hurry or lack of interest or motivation. Secondly, participants were instructed to pass the question if the reimbursement reform had had no effect, so presumably some passed the question because the reimbursement reform had not truly affected them. Copayment increased no more than slightly among some patients, for example those using only metformin, and it is possible that a minor increase in copayment does not impact personal life.

Medicine user charges have been high in Finland compared to other western European countries [27]. According to a Finnish survey conducted in 2010, 11% of the respondents did not fill a prescription due to cost within the past year [28]. High user charges as well as reimbursement reforms can hinder patients’ economic possibilities to purchase medicines and decreasing the use of antidiabetic medicines can be especially detrimental because diabetes with poor glycemic control can lead to serious complications. Before the implementation of the reimbursement reform there was discussions that it might lead to impaired therapeutic control or increased use of insulins among patients with type 2 diabetes [7]. According to our study, however, the reimbursement reform did not seem to affect patients’ therapeutic control largely because only a few reported that it had impaired. Moreover, participants reported effects on the use of medicines fairly seldom: 14 participants reported that they had discontinued using a medicine and only a few participants had switched to insulin, which remained at the highest rate of reimbursement. This accords with the overall consumption of antidiabetic medicines in Finland after the reform [10]. It is also in line with previous register-based studies in other countries, in which the consumption of oral antidiabetic medicines, for which reimbursement was restricted or reduced, did not decrease significantly after the reimbursement reform [18, 20].

According to our logistic regression, income, work status or education level did not affect the experience of the reimbursement reform. In other words, it seems the reimbursement reform has affected all socio-economic classes. The use of combinations of oral blood glucose lowering medicines, glitazones, DPP-4-inhibitors, GLP-1-analogues and SGLT2-inhibitors at baseline were associated with reporting an effect. This result is logical because above-mentioned medicines are expensive, so their copayments increased substantially. It also accords with reports on SGLT2-inhibitors, GLP-1-analogues and glitazones being bought more often with income support in 2017 [11]. Early diabetes reduced the odds of reporting an effect. Those who had had diabetes for long might be attuned to certain priced antidiabetic medicines, thus it is possible that the reimbursement reform affects them more. Moreover, participants not having the annual deductible exceeded were more likely to report an effect, which is sensible. If the annual deductible has exceeded already before the reform, it perhaps does not carry much significance if the limit exceeds faster due to the reimbursement reform.

This study has a number of strengths. Our study provides information about the effects of the reimbursement reform from the patients’ point of view. Nearly every participant used other antidiabetic medicines than insulins and therefore the reimbursement reform influenced particularly them. We gained information from medium-sized population since we had 603 participants. Our results should be generalizable to patients with type 2 diabetes in Finland because we had participants across the country and medication use was quite similar to Finnish patients with type 2 diabetes, although middle-aged patients seemed to be overrepresented and patients 80-years-old and older seemed to be underrepresented. Our study has also some limitations. Participants were recruited at pharmacies across Finland using convenience sampling. It is however unknowable if pharmacists tried to recruit every customer who met the inclusion criteria and what kind of customers refused to participate in this study. It is also possible, that participants in the 12-month follow-up survey had experienced more effects than patients dropping out. Most of the data were collected by phone interviews which involves a risk of the interviewer leading the participants or misconstruing the answers. Phone interviews were performed by several interviewers. Some of the answers collected by phone interviews suggest that some interviewers possibly had asked “has the reimbursement reform affected” instead of “how has the reimbursement reform affected”. It is also possible that some participants exaggerated the effects of reimbursement reform since the reimbursement reform raised strong objections among some Finns [29].

## Conclusions

Almost half of the participants reported an effect, most commonly economic effects, caused by the reimbursement reform of antidiabetic medicines. Socio-economic status was not associated with the likelihood of reporting an effect. To our best knowledge, this was the first study which evaluated the effects of a reimbursement reform from the patients’ point of view. Moreover, this study showed that the effects of reimbursement reforms can be studied by means of a survey. The effects of reimbursement reforms must be evaluated widely as all effects are not captured through register data, so more studies are needed from the patients’ perspective.

## Supplementary information

**Additional file 1.** Baseline characteristics of patients dropping out of the 12-month follow-up survey and the survey participants.

**Additional file 2.** Age groups of survey participants and Finns entitled to reimbursement for other antidiabetic medicines than insulin in 2016.

**Additional file 3.** Medication use at baseline among survey participants and Finnish recipients of reimbursement for antidiabetic medicines other than insulin in 2016.

## Data Availability

The data that support the findings of this study are available from Farenta Oy but restrictions apply to the availability of these data, which were used under license for the current study, and so are not publicly available. Data are however available from the authors upon reasonable request and with permission of Farenta Oy.

## Abbreviations

CI: Confidence interval
DPP-4: Dipeptidyl peptidase-4
GLP-1: Glucagon-like peptide-1
OR: Odds ratio
SD: Standard deviation
SGLT2: Sodium-glucose co-transporter 2
