# Supplementary material for: The effects of reimbursement reform of antidiabetic medicines from the patients’ perspective – a survey among patients with type 2 diabetes in Finland
Source: BMC Health Serv Res. 2019 Oct 29;19:769. doi: 10.1186/s12913-019-4633-9 (PMC6819478; doi:10.1186/s12913-019-4633-9)
Supplement: Supplementary file 1 — Additional file 1. Baseline characteristics of patients dropping out of the 12-month follow-up survey and the survey participants. [file 12913_2019_4633_MOESM1_ESM.docx]

Additional file 1. Baseline characteristics of patients dropping out of the 12-month follow-up survey and the survey participants

|  | **Patients dropping out (n=352)**  **% (n)** | **Participants**  **(n=603)**  **% (n)** |
| --- | --- | --- |
| **Sociodemographic and -economic variables** | | |
| **Mean age**, years (SD) | 64.6 (10.6) | 65.5 (10.0) |
| **Female gender** | 47.4 (167) | 48.6 (293) |
| **Household’s monthly income** | | |
| Less than EUR 1 000 | 11.1 (39) | 11.3 (68) |
| EUR 1 000–1 999 | 39.2 (138) | 37.1 (224) |
| EUR 2 000–2 999 | 23.0 (81) | 28.7 (173) |
| EUR 3 000–3 999 | 11.4 (40) | 11.3 (68) |
| EUR 4 000 or more | 15.3 (54) | 11.6 (70) |
| **Education** | | |
| Basic education or some other | 39.8 (140) | 39.6 (239) |
| Vocational upper secondary education and training | 17.3 (61) | 20.6 (124) |
| Post-secondary non-higher vocational education | 24.1 (85) | 22.7 (137) |
| Matriculation examination | 7.1 (25) | 5.6 (34) |
| University or polytechnic degree | 11.6 (41) | 11.4 (69) |
| **Work/life situation*** | | |
| Working | 22.2 (78) | 16.4 (99) |
| Not working or outside working life | 77.8 (274) | 83.6 (504) |
| **Financial difficulties in purchasing antidiabetic medicines** | 14.5 (51) | 17.7 (107) |
| **Annual maximum limit on out-of-pocket costs exceeded** | | |
| Yes | 13.9 (49) | 12.3 (74) |
| Will be exceeded | 5.4 (19) | 6.1 (37) |
| Will not likely be exceeded or does not know | 80.7 (284) | 81.6 (492) |
| **Diabetes-related variables** | | |
| **How long has had diabetes** | | |
| More than 20 years | 8.5 (30) | 10.0 (60) |
| 11–20 years | 25.9 (91) | 26.9 (162) |
| 6–10 years | 32.7 (115) | 32.7 (197) |
| 2–5 years | 24.7 (87) | 24.0 (145) |
| 1 year or less | 8.2 (29) | 6.5 (39) |
| **Mean number of diabetes complications** (SD) | 0.7 (1.1) | 0.8 (1.1) |
| **Use of insulin** | 27.6 (97) | 30.8 (186) |
| **Use of other antidiabetic medicines than insulin** | 96.9 (341) | 97.5 (588) |
| Metformin | 76.7 (270) | 75.8 (457) |
| Sulfonylureas | 3.1 (11) | 3.6 (22) |
| Combination of oral blood glucose lowering medicines | 11.1 (39) | 12.4 (75) |
| Glitazones | 3.1 (11) | 2.7 (16) |
| DPP-4-inhibitors | 34.9 (123) | 36.8 (222) |
| Glinides | 0.6 (2) | 1.0 (6) |
| GLP-1-analogues | 8.8 (31) | 9.5 (57) |
| SGLT2-inhibitors | 12.8 (45) | 14.1 (85) |
| **Use of hypertension medication** | 80.7 (284) | 79.6 (480) |
| **Use of cholesterol medication** | 66.8 (235) | 67.7 (408) |

*statistically significant difference (p<0.05) between patients dropping out and survey participants

DPP-4: Dipeptidyl peptidase-4; GLP-1: Glucagon-like peptide-1; SD: Standard deviation; SGLT2: Sodium-glucose co-transporter 2
