# Supplementary material for: The effects of reimbursement reform of antidiabetic medicines from the patients’ perspective – a survey among patients with type 2 diabetes in Finland
Source: BMC Health Serv Res. 2019 Oct 29;19:769. doi: 10.1186/s12913-019-4633-9 (PMC6819478; doi:10.1186/s12913-019-4633-9)
Supplement: Supplementary file 2 — Additional file 2. Age groups of survey participants and Finns entitled to reimbursement for other antidiabetic medicines than insulin in 2016. [file 12913_2019_4633_MOESM2_ESM.docx]

Additional file 2. Age groups of survey participants at baseline and Finns entitled to reimbursement for other antidiabetic medicines than insulin in 2016 (Statistical database Kelasto 2019)

| Age group (years) | Survey participants (n=603)  % (n) | People entitled to reimbursement for other antidiabetic medicines than insulin  (n=324 618)  % (n) |
| --- | --- | --- |
| <40 | 2.3 (14) | 7.4 (24 032) |
| 40–49 | 4.8 (29) | 6.6 (21 413) |
| 50–59 | 18.7 (113) | 15.9 (51 590) |
| 60–69 | 43.4 (262) | 29.1 (94 557) |
| 70–79 | 26.2 (158) | 25.9 (84 081) |
| ≥80 | 4.5 (27) | 15.1 (48 945) |
