# Supplementary material for: The effects of reimbursement reform of antidiabetic medicines from the patients’ perspective – a survey among patients with type 2 diabetes in Finland
Source: BMC Health Serv Res. 2019 Oct 29;19:769. doi: 10.1186/s12913-019-4633-9 (PMC6819478; doi:10.1186/s12913-019-4633-9)
Supplement: Supplementary file 3 — Additional file 3. Medication use at baseline among survey participants and Finnish recipients of reimbursement for antidiabetic medicines other than insulin in 2016. [file 12913_2019_4633_MOESM3_ESM.docx]

Additional file 3. Medication use at baseline among survey participants and recipients of reimbursement for antidiabetic medicines other than insulin in 2016 (Statistical database Kelasto 2019)

| Medication group (ATC code) | Survey participants (n=603)  % (n) | Recipients of reimbursement for antidiabetic medicines other than insulin  (n=307 885)  % (n) |
| --- | --- | --- |
| Metformin (A10BA02) | 75.8 (457) | 78.3 (240 921) |
| Sulfonylureas (A10BB) | 3.6 (22) | 4.0 (12 261) |
| Combinations of oral blood glucose lowering drugs (A10BD) | 12.4 (75) | 12.8 (39 367) |
| Thiazolidinediones (A10BG) | 2.7 (16) | 2.0 (6 265) |
| Dipeptidyl peptidase 4 (DPP-4) inhibitors (A10BH) | 36.8 (222) | 31.4 (96 819) |
| Glucagon-like peptide-1 (GLP-1) analogues (A10BJ) | 9.5 (57) | 4.9 (15 190) |
| Sodium-glucose co-transporter 2 (SGLT2) inhibitors (A10BK) | 14.1 (85) | 8.3 (25 560) |
| Other blood glucose lowering drugs, excl. insulins (A10BX) | 1.0 (6) | 0.7 (2 166) |
